# The effect of minimally invasive intracerebral haematoma evacuation on early perihaematomal oedema formation

**DOI:** 10.1093/esj/aakaf025

**Published:** 2026-01-01

**Authors:** Maaike P Cliteur, Lotte Sondag, Sjoert A H Pegge, Wilmar M T Jolink, D Verbaan, Hieronymus D Boogaarts, Marieke J H Wermer, Ruben Dammers, Floris H B M Schreuder, Catharina J M Klijn

**Affiliations:** Department of Neurology, Donders Institute for Brain, Cognition and Behaviour, Radboud University Medical Center, Nijmegen, The Netherlands; Department of Neurology, Donders Institute for Brain, Cognition and Behaviour, Radboud University Medical Center, Nijmegen, The Netherlands; Department of Radiology, Radboud University Medical Center, Nijmegen, The Netherlands; Department of Neurology and Neurosurgery, University Medical Center Utrecht Brain Center, Utrecht University, Utrecht, The Netherlands; Department of Neurosurgery, Amsterdam University Medical Center, University of Amsterdam, Amsterdam, The Netherlands; Department of Neurosurgery, Radboud University Medical Center, Nijmegen, The Netherlands; Department of Neurology, Leiden University Medical Center, Leiden, The Netherlands; Department of Neurosurgery, Erasmus Medical Center, Erasmus MC Stroke Centre, Rotterdam, The Netherland; Department of Neurology, Donders Institute for Brain, Cognition and Behaviour, Radboud University Medical Center, Nijmegen, The Netherlands; Department of Neurology, Donders Institute for Brain, Cognition and Behaviour, Radboud University Medical Center, Nijmegen, The Netherlands

**Keywords:** intracerebral haemorrhage, perihaematomal oedema, surgery

## Abstract

**Introduction:**

In patients with intracerebral haemorrhage (ICH), perihaematomal oedema (PHO) is considered a marker of secondary injury and is associated with worse functional outcomes. Minimally invasive surgery (MIS) has been suggested to reduce PHO when performed within 72 h of symptom onset. However, the effect of early surgery on PHO formation remains unclear. We aimed to determine the effect of MIS within 8 h of ICH onset on PHO formation.

**Patients and methods:**

We included patients with spontaneous, supratentorial ICH ≥10 mL undergoing MIS within 8 h from the DIST-pilot study and compared them to patients receiving standard care from a cohort study. ICH and PHO volumes at baseline and 24(±12) h were manually segmented. The primary outcome was absolute PHO (aPHO) volume at 24 h. Secondary outcomes included aPHO growth between baseline, and 24 h and oedema extension distance (OED).

**Results:**

We included 34 patients (median age 61 years, 68% male) undergoing MIS and 16 patients (median age 65 years, 69% male) receiving standard medical care. At baseline, median ICH, aPHO and OED volume were similar between groups. Median aPHO volume at 24 h was similar between groups (median difference −3.0 mL, 95% CI, −19.4 to 9.8, *P* =.67), while median aPHO growth was smaller in the MIS-group (median difference −6.8 mL, 95% CI, −18.67 to 0.33, *P* =.002). Median OED was greater in the MIS-group (median difference 0.18 cm, 95% CI, 0.05–0.40, *P* =.002).

**Conclusion:**

Absolute PHO growth in the first 24 h after ICH was less pronounced after early MIS than after standard care, suggesting that early MIS may ameliorate secondary injury after ICH.

## Graphical Abstract

**Graphical abstract:**
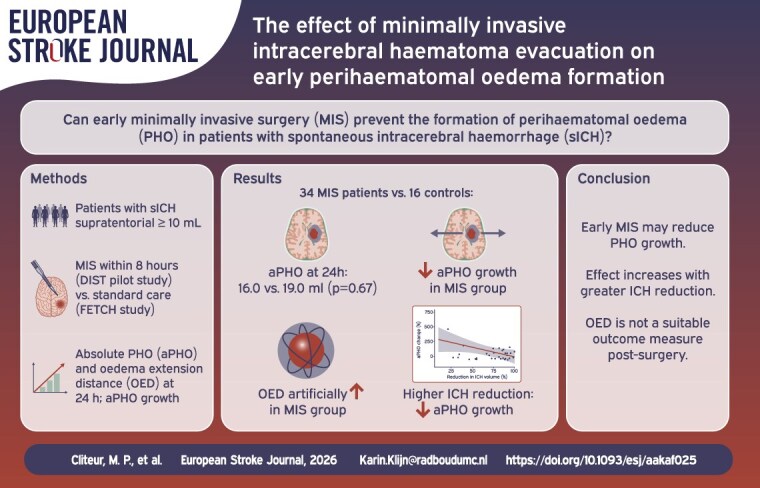

## Introduction

Despite advancements in medical care and research, spontaneous intracerebral haemorrhage (ICH) remains the most lethal subtype of stroke.^1^^,^^2^ Brain injury in ICH occurs as a result of the direct mass impact of the haematoma formation, but also develops in the hours after ICH as a consequence of the neuroinflammatory cascade driven by toxic and plasma derived components that affect the surrounding brain parenchyma.^3–6^ Perihaematomal oedema (PHO) is considered a radiological marker of secondary injury, strongly correlates with ICH volume and is an independent predictor of functional outcome.^7^^,^^8^

Minimally invasive surgery (MIS) has emerged as a promising technique for haematoma evacuation in patients with ICH.^9^ The ENRICH trial was the first to demonstrate that MIS performed within 24 h of onset improves functional outcomes in patients with spontaneous ICH of 30 to 80 mL.^10^ In ENRICH, the presence of PHO was less frequent in the MIS-group, supporting the hypothesis that haematoma reduction by MIS may attenuate the inflammatory cascade leading to PHO formation. These findings are in accordance with previous studies that have shown that surgical ICH removal within 72 h after symptom onset can decrease PHO volumes during the first week.^11–16^

In recent years, it has been suggested that earlier haematoma evacuation, within the first hours after onset, may offer greater benefit than delayed interventions.^17^ As the neuroinflammatory cascade starts in the first hours after ICH onset,^5^^,^^18^ MIS in the early timeframe (within 8 h of symptom onset) might attenuate secondary injury more effectively than MIS performed within 24 to 72 h. However, to date no studies have evaluated the effect of MIS within 8 h on the development of PHO.

We aimed to determine the effect of minimally invasive surgical haematoma evacuation within 8 h of symptom onset on PHO volume at 24 h. We hypothesise that early effective minimally invasive haematoma removal will prevent early PHO formation. Additionally, we aimed to determine the most informative metric to quantify PHO after MIS.

## Patients and methods

We conducted a retrospective analysis of prospectively collected data from 2 sources: the Dutch Intracerebral haemorrhage Surgery Trial (DIST) pilot study^19^ and the Finding Etiology of spontaneous Cerebral Haemorrhage (FETCH) study.^20^ Both studies were approved by their respective local Medical Ethics Committees. All participants or their legal representatives provided written informed consent.

**Figure 1:**
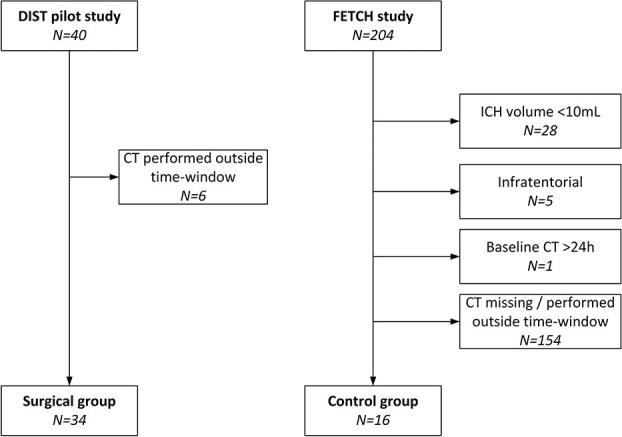
Patient selection. Abbreviations: CT = Computed tomography scan; ICH = Intracerebral haemorrhage.

### Subjects

The DIST pilot study was a multicentre, prospective, non-randomised study investigating the safety and technical efficacy of minimally invasive endoscopy-guided surgical evacuation of spontaneous supratentorial ICH within 8 h after symptom onset. The methods have been described previously.^19^ For the current analysis, we included patients who underwent minimally invasive endoscopy-guided haematoma evacuation, had an available CT scan at the time of admission, and a postoperative CT scan obtained 24 (±12) h after the initial CT, allowing for assessment of PHO development. Because control patients from the DIST pilot study did not undergo a CT scan at 24 h, we selected a comparator group of patients receiving standard medical management from the FETCH study. This multicentre prospective cohort study included 204 consecutive adults with spontaneous ICH in 3 Dutch hospitals between October 2013 and December 2018. The inclusion and exclusion criteria have been described previously.^20^ Both studies followed the Dutch ICH guidelines for the non-surgical management of ICH, which included admission to a specialised stroke or intensive care unit, intensive neurological monitoring and blood pressure control. For the present analysis, we selected patients from the FETCH study with a spontaneous, supratentorial ICH of ≥10 mL, with a CT scan obtained within 1 day of symptom onset, and a second CT scan at 24 (±12) h after the baseline CT.

For all included patients, we retrieved clinical data including age, sex, medical history, date and time of symptom onset, Glasgow Coma Scale (GCS) and NIHSS scores at admission.

### Radiological assessment

Baseline and follow-up non-contrast CT scans were manually segmented by a central assessor (MC, 6 years of experience in rating acute ICH imaging) to determine ICH and PHO volumes using open source segmentation software ITK-SNAP (version 3.8.0, Cognitica).^21^ A second rater (SP, neuroradiologist with 21 years of experience) independently rated a sample of 25% of the scans to determine interrater reliability. ICH and PHO volume at 24 h were assessed blinded for the baseline scans. PHO was defined as the hypodense rim surrounding the ICH with Hounsfield unit values between 5 and 33.^22^ ICH and PHO volume were calculated using ITK-SNAP based on voxel volume. ICH location was classified as supratentorial deep (basal ganglia or thalamus) or lobar, using the CHARTS instrument.^23^

The primary outcome was absolute PHO (aPHO) volume at 24 h. Secondary outcomes were (1) aPHO growth, defined as change in aPHO volume from baseline to 24 h; (2) oedema extension distance (OED) at 24 h. The OED reflects the distance of the ICH edge to the outer border of PHO, assuming a spherical, fairly consistent inflammatory response along the haematoma parameter. It is calculated using the formula OED = $\sqrt[3]{\frac{PHE\ vol+ ICH\ vol}{\frac{4}{3\ }\ \pi }}$—$\sqrt[3]{\frac{ICH\ vol}{\frac{4}{3\ }\ \pi }}$^24^ (3) relative pericavital oedema (PCO), defined as the ratio of aPHO at follow-up divided by baseline ICH volume.^16^

### Statistical analysis

Data are presented as either mean ± SD or median with IQR depending on normality of distribution. Differences in baseline characteristics were tested with Mann–Whitney U tests for continuous parameters and Fisher’s exact test for categorial data. Standard box plots were constructed to visually display distributions of ICH and PHO volumes. Interrater reliability for volumetric segmentation was assessed using the intraclass correlation coefficient (ICC). An ICC value < 0.50 was classified as poor, 0.50–0.75 as moderate, 0.75–0.90 as good, and >0.90 as excellent reliability.^25^ Wilcoxon rank-sum tests were used to determine PHO differences between groups. Ninety-five percent CIs of the observed median differences between the groups were estimated through bootstrap resampling with 5000 iterations. Additionally, the primary and secondary analyses (except for PCO) were adjusted for baseline ICH volume using analysis of covariance. Linear regression models with log-transformed outcomes were fitted to account for non-normal distributions, providing group ratios with 95% CI and corresponding p-values. In the MIS group, we assessed the association between the percentage of ICH reduction and percentual aPHO change, and OED at 24 h with univariable linear regression. Subsequently, we constructed a multivariable linear regression model adjusting for baseline ICH volume as a predictor for PHO. All statistical analyses were performed using R (version 4.1.3, R Foundation for Statistical Computing, Vienna, Austria) using packages “tidyverse,” “dplyr,” “ggplot2,” “pracma,” “psych,” “broom,” “boot” and “lme4.” Statistical significance was set at *P* <.05.

## Results

We included 34 patients that underwent MIS (median age 61 years, IQR 53–67) and 16 patients who received standard medical care (median age 65 years, IQR 51–75) (Figure 1). In the MIS group, GCS at admission was significantly lower, and NIHSS scores significantly higher, compared to those receiving standard care. Time since symptom onset to baseline NCCT was similar between groups (median 3.9 h in the MIS-group vs 4.0 h in controls, *P* =.25) (Table 1).

**Table 1:** Patient and imaging characteristics

	**Surgical (DIST) *n* = 34**	**Control (FETCH) *n* = 16**	***P*-value**
**Clinical characteristics**			
Age (median, IQR)	61 (53–67)	65 (51–75)	.52
Male (*n*, %)	23 (67.6%)	11 (68.8%)	1.00
Hypertension (*n*, %)	13 (38.2%)	10 (62.5%)	.14
Atrial fibrillation (*n*, %)	3 (9.9%)	2 (12.5%)	.65
Diabetes mellitus (*n*, %)	5 (14.7%)	4 (25.0%)	.44
GCS at admission (median, IQR)	12 (9–14)	14 (13–15)	.02
NIHSS at admission (median, IQR)	19 (13–21)	13 (6–15)	<.01
**Baseline CT scan**			
Interval between symptom onset and CT, h (median, IQR)	3.9 (2.4–5.6)	4.0 (2.7–11.7)	.25
ICH location	Deep 24 (71%) Lobar 10 (29%)	Deep 9 (56%) Lobar 7 (44%)	.35
IVH present (*n*, %)	15 (44.1%)	9 (56.3%)	.55
ICH volume at admission, mL (median, IQR)	41.5 (28.2–61.8)	31.3 (12.5–52.4)	.17
aPHO volume at admission, mL (median, IQR)	15.7 (8.6–30.6)	5.5 (2.3–24.1)	.11
rPHO volume at admission (median, IQR)	0.40 (0.21–0.63)	0.28 (0.13–0.47)	.18
OED at admission, cm (median, IQR)	0.23 (0.15–0.35)	0.12 (0.08–0.29)	.12
**Follow-up CT scan**			
Interval between symptom onset and start MIS, h (median, IQR)	7.1 (5.7–8.2)	n.a.	-
Interval between CTs, h (median, IQR)	26.2 (23.7–29.6)	25.9 (22.9–29.7)	.51
ICH volume second CT, mL (median, IQR)	8.4 (3.2–18.8)	30.3 (15.0–58.1)	<.001
aPHO volume second CT, mL (median, IQR)	16.0 (11.2–36.9)	19.0 (8.4–35.4)	.67
rPHO volume second CT (median, IQR)	2.0 (1.0–3.2)	0.58 (0.43–0.97)	<.001
OED second CT, cm (median, IQR)	0.51 (0.41–0.77)	0.32 (0.23–0.43)	.002
**Interval changes**			
Difference ICH volume, mL (median, IQR)	−30.5 (−44.0 to −19.6)	0.69 (−0.9 to 3.5)	<.001
Difference aPHO volume, mL (median, IQR)	2.7 (−7.4 to 7.9)	9.5 (4.0–19.7)	.002
Percentage difference aPHO (median IQR)	17.3 (−33.6 to 96.7)	141.5 (54.3–319.1)	.006
Difference rPHO volume (median IQR)	1.5 (0.7–3.0)	0.32 (0.19–0.53)	<.001
Difference in OED, cm (median IQR)	0.25 (0.12–0.53)	0.18 (0.10–0.24)	.14
Relative PCO (median IQR)	0.43 (0.27–0.57)	0.62 (0.42–0.94)	.03

Abbreviations: aPHO = absolute perihaematomal oedema; CT = computed tomography; GCS = Glasgow Coma Scale; ICH = intracerebral haemorrhage; IVH = intraventricular haemorrhage; IQR = interquartile range; n.a. = not applicable; NIHSS = National Institute of Health Scale score; OED = oedema extension distance; rPHO = relative perihaematomal oedema; PCO = pericavital oedema.

Median ICH volume at admission was not significantly different in the MIS-group (*P* =.17), as were aPHO (*P* =.11), rPHO (*P* =.18) and OED (*P* =.12) (Table 1). Inter-rater agreement for ICH and PHO was excellent, with an ICC of 0.98.

Median time from symptom onset to start of MIS was 7.1 h (IQR 5.7–8.2 h). Compared to baseline ICH volume, median ICH volume at 24 h was significantly reduced in the group that underwent MIS (median difference −30.5 mL, 95% CI, −37.7 to −24.2, *P* <.01) whereas the median ICH volume remained similar in the patients receiving standard medical care (median difference −0.69 mL, 95% CI, −3.17 to 0.84, *P* =.27). Median aPHO volume at 24 h was similar between groups (median difference −3.0 mL, 95% CI, −19.4 to 9.8, *P* =.67) and this remained unchanged after correction for baseline ICH volume (adjusted group ratio = 0.80, 95% CI, 0.48–1.32, *P* =.37). However, aPHO growth was smaller in the MIS-group (median difference −6.8 mL, 95% CI, −18.67 to 0.33, *P* =.002). This finding persisted after adjustment for baseline ICH volume (adjusted group ratio = 0.53, 95% CI, 0.31–0.92, *P* =.02), confirming lower aPHO growth in the MIS group compared to controls. In contrast, median OED at 24 h was larger in the MIS-group compared to controls at 24 h in both unadjusted (median difference 0.18 cm, 95% CI, 0.05–0.40, *P* =.002) and adjusted analysis (adjusted group ratio 1.79, 95% CI, 1.19–2.67, *P* =.01). Relative PCO volume was lower in the MIS group than in controls (median difference −0.19, 95% CI, −0.54 to 0.03, *P* =.03).

**Figure 2:**
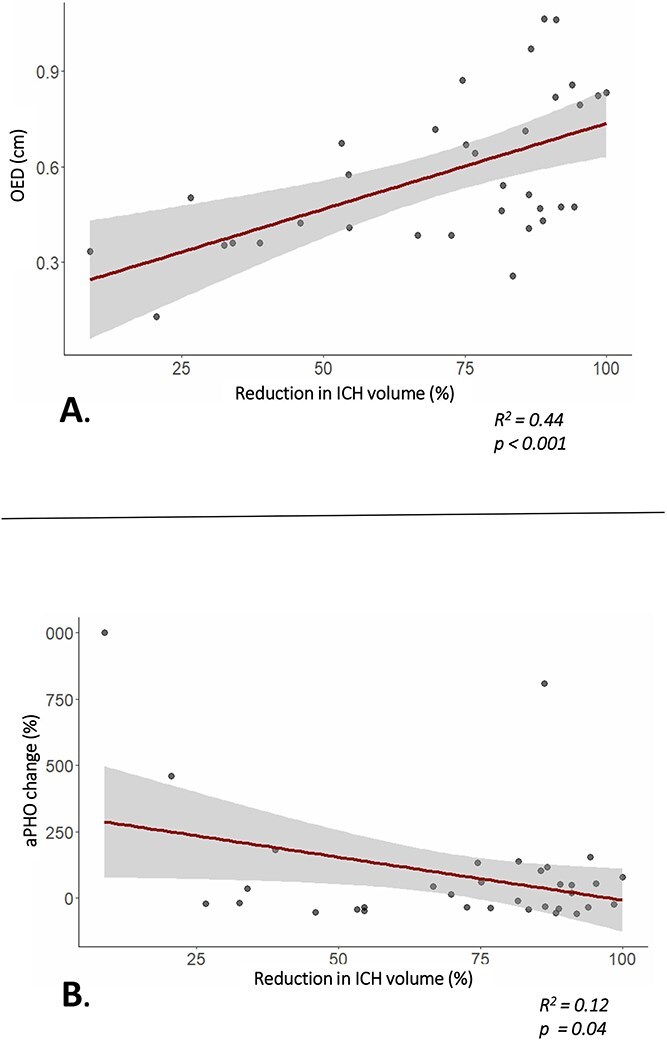
Effect of percentual ICH reduction in patients treated with MIS. (A) Association between percentual ICH reduction and OED at 24 h; (B) association between percentual ICH reduction and change of aPHO from baseline to follow-up at 24 h. Abbreviations: aPHO = Absolute perihaematomal oedema; ICH = Intracerebral haemorrhage; OED = Oedema extension distance; rPHO = Relative perihaematomal oedema. Shaded area indicates 95% CI.

In the MIS group, larger surgical ICH reduction was associated with a larger OED at 24 h (β = 0.054 per 10% ICH reduction, 95% CI, 0.026–0.082, *R*^2^ = 0.44, *P* = < .001, Figure 2). Greater ICH reduction was associated with a lower percentual change in aPHO between baseline and 24 h (β = −32.3 per 10% ICH reduction, 95% CI, −63.7 to −1.0, *R*^2^ = 0.12, *P* = .04, Figure 2). This association persisted after additional adjustment for baseline ICH volume, though it was no longer statistically significant (β = −32.3 per 10% ICH reduction, 95% CI, −63.7 to −1.0, *R*^2^ = 0.14, *P* =.08).

## Discussion

In this study, minimally invasive hematoma evacuation within 8 h of symptom onset was associated with reduced aPHO growth within 24 h post-surgery. This association was modified by the achieved ICH volume reduction. These data suggest that early MIS may ameliorate secondary injury after ICH, and that this effect is more prominent when a larger percentage of the haematoma is removed.

Our findings are in accordance with the results in patients who were operated on at later timepoints in the ENRICH^10^ and MISTIE II^14^ trials. In the ENRICH trial, patients were treated at a median of 17 h after symptom onset, while in MISTIE III, surgery was delayed at least 6 h after onset to confirm stable haematoma volume, resulting in treatment commencing at a mean of 58 h after ICH onset.^10^^,^^26^ In contrast, MIS was performed at a median of 7 h since symptom onset in the DIST pilot study. Considering the proposed time- and dose-dependent toxic effects of the haematoma on surrounding brain tissue,^16^ early interventions may offer a greater therapeutical potential in limiting secondary brain injury than later interventions. With our current analysis, we provide the first evidence indicating that ultra-early MIS can indeed prevent early PHO formation. In addition, our results indicate that the effect of haematoma evacuation on oedema growth may increase when a larger percentage of the haematoma is removed.

In contrast to the reduced aPHO growth, we found a marked increase in the calculated OED at 24 h in the MIS-group. This increase in OED was driven by the percentual ICH volume reduction, suggesting that the OED formula may not be suitable post-surgery. The assumption of a spherical ICH shape may no longer hold true in patients after MIS. In addition, successful haematoma evacuation reduces the ICH radius substantially. Since OED is defined as the difference between the radii of a sphere encompassing both PHO and ICH and of a sphere containing only ICH, a decrease in ICH volume leads to a greater reduction in the ICH radius compared to the ICH plus PHO radius. This results in the erroneous impression that the oedema (measured as OED) increases after MIS. OED has been shown to be a robust outcome measure for trials aimed at PHO formation primarily.^24^ However, our findings demonstrate that OED is not a reliable outcome metric in trials targeting haematoma volume reduction.

Our study has several strengths. First, we studied MIS performed within 8 h, offering new insights into the pathophysiological impact of early haematoma reduction on the development of secondary brain injury. While several ongoing trials are investigating MIS in early time windows, data examining the effects of haematoma evacuation within the first 8 h of symptom onset are not yet available. Second, we achieved a good median ICH reduction of 82% in the MIS group, allowing for a reliable assessment of the effect of ICH volume reduction on PHO formation. Third, we minimised reporting bias by assessing CT scans at 24 h blinded for the assessment of the baseline scan.

Our study also has limitations. First, the CT imaging used to quantify PHO was performed at 24 h, while PHO increases over the first days with a reported peak at approximately 1 to 2 weeks after ICH onset.^5^^,^^27^ However, most PHO growth occurs in the first 24–48 h^16^ and previous studies have found that the PHO expansion rate within the first 24 h is a significant predictor for functional outcome and mortality.^27^ Thus, reducing PHO formation in this early time window may therefore have a beneficial impact on neurological outcomes in patients treated with MIS. Moreover, early assessment of PHO development in an early phase after haematoma removal offers a direct evaluation of the impact of the procedure, eliminating other possible influencing factors that might occur during the following days. Two other important limitations of this study are the retrospective nature and the use of patient data from 2 different studies. Although this design allowed us to explore PHO dynamics across a unique population of minimally invasive and conservatively treated patients, it carries an inherent risk of selection bias and between-study variability. An additional limitation is the relatively small sample size, particularly in the control group, which may impact the study precision. Lastly, the high level of selection introduces the risk of bias and may limit the generalisability of our findings. The ongoing DIST RCT (NCT05460793), which includes CT imaging at day 1 and 6 post-surgery, will provide an opportunity to validate our findings in a larger, prospective cohort at multiple timepoints.

Considering the association of PHO with functional outcome,^8^^,^^28^ our results emphasise the importance of assessing PHO formation in trials aimed at (minimally invasive) surgical haematoma evacuation. Standardising PHO measurement in these trials will help to better understand the effect of surgical treatment on secondary brain injury and its impact on the therapeutic potential of MIS in ICH. Our findings indicate that aPHO growth is the most informative method to measure PHO post-surgery, while OED does not accurately reflect PHO development in surgically treated patients.

In conclusion, we found that MIS within 8 h of ICH onset is associated with reduced growth of aPHO volume at 24 h, indicating that early haematoma evacuation can limit early secondary brain injury. Future trials investigating surgical intervention in ICH should include PHO growth as a key outcome measure and avoid reliance on OED in the postoperative setting.
